# Evaluating the association between the introduction of mandatory calorie labelling and energy consumed using observational data from the out-of-home food sector in England

**DOI:** 10.1038/s41562-024-02032-1

**Published:** 2024-11-25

**Authors:** Megan Polden, Andrew Jones, Michael Essman, Jean Adams, Tom R. P. Bishop, Thomas Burgoine, Stephen J. Sharp, Martin White, Richard Smith, Aisling Donohue, Rozemarijn Witkam, I. Gusti Ngurah Edi Putra, Jane Brealey, Eric Robinson

**Affiliations:** 1 Department of Primary Care and Mental Health, University of Liverpool, Liverpool, UK; 2 NIHR Applied Research Collaboration, North West Coast, Liverpool, UK; 3 Lancaster University, Health Research, Lancaster, UK; 4 Liverpool John Moores University, Liverpool, UK; 5 MRC Epidemiology Unit, University of Cambridge, Cambridge, UK; 6 Faculty of Health and Life Sciences, University of Exeter, Exeter, UK; 7 Department of Psychology, University of Liverpool, Liverpool, UK

**Keywords:** Human behaviour, Health policy

## Abstract

In April 2022, mandatory kilocalorie (kcal) labelling in the out-of-home food sector was introduced as a policy to reduce obesity in England. Here we examined whether the implementation of this policy was associated with a consumer behaviour change. Large out-of-home food sector outlets subject to kcal labelling legislation were visited pre- and post-implementation, and customer exit surveys were conducted with 6,578 customers from 330 outlets. Kcals purchased and consumed, knowledge of purchased kcals and reported noticing and use of kcal labelling were examined. The results suggested that the introduction of the mandatory kcal labelling policy in England was not associated with a significant decrease in self-reported kcals purchased (*B* = 11.31, *P* = 0.564, 95% confidence interval (CI) −27.15 to 49.77) or consumed (*B* = 18.51, *P* = 0.279, 95% CI −15.01 to 38 52.03). Post-implementation, participants underestimated the energy content of their purchased meal less (*B* = 61.21, *P* = 0.002, 95% CI 21.57 to 100.86) and were more likely to report noticing (odds ratio 2.25, *P* < 0.001, 95% CI 1.84 to 2.73) and using (odds ratio 2.15, *P* < 0.001, 95% CI 1.62 to 2.85) kcal labelling, which may have wider public health implications.

## Abstract (web summary)

The introduction of mandatory calorie labelling in out-of-home food outlets in England was not associated with a reduction in calories purchased or consumed. There was a post-policy increase in self-reported noticing and use of calorie labelling.

## Main

Food provided in the OHFS tends to be energy-dense and high in kilocalories (kcals)^1,2^. Frequently consuming food from the OHFS is associated with increased obesity risk^3^. This is problematic because, according to a 2015 study, 27% of UK adults eat foods in the OHFS once per week or more^4^. Obesity is a major global public health problem. In England, recent data suggest that 26% of people live with obesity^5^, with obesity linked to a range of diseases, including type 2 diabetes, several cancers and cardiovascular disease^6–10^. Obesity produces a substantial health care burden in the United Kingdom^11,12^. A likely contributing factor to obesity is the out-of-home food sector (OHFS). Obesity is also socio-demographically patterned^13,14^, and public health interventions are required to reduce obesity and its social inequalities.

Multiple countries, including the United States^15^ and parts of Canada^16^ have implemented mandatory kcal labelling legislation in response to the growing contribution of the OHFS on diet. In 2011, as part of the UK public health responsibility deal^17^, OHFS businesses were encouraged to make voluntary pledges to provide kcal labelling^18^. However, a 2018 study found that only 17% (18 out of 104) of OHFS outlets assessed in England were providing in-store kcal labelling, and when labelling was present, it did not adhere to government proposed best practice guidelines^19^. Motivated by a lack of voluntary compliance, the UK government announced the Calorie Labelling (Out of Home Sector) (England) Legislation 2021, with a policy enactment deadline of 6 April 2022 for eligible businesses^20–22^. The legislation applied to large (>250 employees) businesses (cafes, fast-food outlets, sit-down restaurants and pubs) in England selling food for immediate consumption. It requires businesses to provide kcal labelling on all unpackaged food and non-alcoholic drink items that are on the menu for more than 30 days per year, alongside contextual information on recommended kcal consumption^23^.

Systematic reviews examining the effect of kcal labelling on consumer behaviour have concluded that kcal labelling has a modest to null effect on kcals selected or purchased^24–29^. For example, a Cochrane review by Crockett et al.^30^ indicated that kcal labelling was associated with a reduction of ~47 kcals purchased^30^, but there was a high level of uncertainty in this estimate. Similar to England, the United States implemented mandatory labelling applying to food outlets with more than 20 locations in 2018. Petimar et al.^31^ examined whether kcal labelling changed purchasing behaviour for meals across 104 restaurants from a fast-food franchise pre- versus post-policy implementation. Using retail transactions, they demonstrated that kcal labelling implementation was associated with a reduction of 54 kcals per transaction^31^.

Kcal labelling in the OHFS could lead to a reduction in kcal consumption by influencing individuals’ food choices^24–29^ and through menu reformulation^24^. In addition, changes in consumer behaviour following the implementation of kcal labelling may be mediated by the level of existing knowledge of the kcal content of menu items in the general population, which may explain varying associations and impacts of kcal labelling in different populations and settings. For example, kcal labelling may have a greater impact on food choices if knowledge of kcal content of OHFS foods is poorer and could also have differing effects depending on whether menu items’ energy content tends to be under- or overestimated. Moreover, it may be the case that people somewhat randomly under- or overestimate the number of kcals in food items. For example, people may underestimate for the least healthy foods but overestimate for the healthiest foods or vice versa. If this is the case, the impact of kcal labelling could be influenced by the consumers’ original assumption of the number of kcals in the food item. Depending on this assumption, kcal labelling may lead to fewer or more kcals purchased by consumers.

So far, there has been no examination of whether the introduction of mandatory kcal labelling in England was associated with a reduction in the amount of energy purchased or consumed from the OHFS. Understanding whether the effects of the introduction of mandatory kcal labelling in the OHFS may differ by population socio-demographics will also be critical to understanding its potential to narrow or widen health inequalities.

This study examined energy purchased and consumed by customers in the OHFS pre- versus post-implementation of mandatory kcal labelling legislation in England. This study also examined whether customer estimation of energy content of their purchases, self-reported noticing and the use of kcal information differed pre- versus post-policy implementation.

**Fig. 1:**
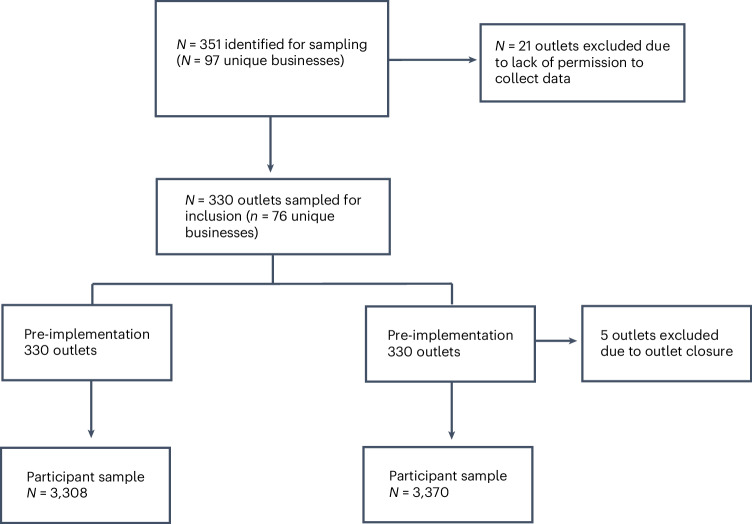
Participant and outlet sample sizes. Sample sizes for pre- and post-implementation customer surveys and reasons for outlet exclusion.

Characteristics of the sampled outlets are reported in Table 1. *N* = 6,578 participants were recruited, *n* = 3,308 pre-implementation and *n* = 3,270 post-implementation (Fig. 1). Across both timepoints, recruited participants were of a similar mean age and a comparable distribution of gender and ethnicity. It should be noted that there was a higher proportion of patients with lower socio-economic position (SEP) in the pre-implementation sample compared with post-implementation. Participant sample information is reported in Table 2. Approximately 28% of participants were recruited from pubs, 25% from restaurants, 24% from fast-food outlets, 20% from cafes and 3% from entertainment venues both pre- and post-implementation.

**Table 1:** Outlet characteristics (outlet type, local authority and LSOA IMD value) for customer exit surveys pre- and post-implementation

	Outlets included pre-implementation (*N* = 330)	Outlets included post-implementation (*N* = 325)
Local authorities *N* (%)		
Liverpool	86 (26%)	84 (26%)
Dudley	84 (25%)	82 (25%)
Milton Keynes	82 (25%)	82 (25%)
Richmond	78 (24%)	77 (24%)
Outlet type *N* (%)		
Cafes	66 (20%)	66 (20%)
Fast food	81 (25%)	80 (25%)
Pubs	92 (27%)	89 (27%)
Restaurants	81 (25%)	80 (25%)
Entertainment	10 (3%)	10 (3%)
LSOA IMD quintiles *N* (%)		
1 (most deprived)	94 (29%)	94 (29%)
2	47 (14%)	44 (14%)
3	59 (18%)	58 (18%)
4	40 (12%)	40 (12%)
5 (least deprived)	90 (27%)	89 (27%)

*N*, number of samples; LSOA, lower layer super output area; IMD, indices of multiple deprivation.

**Table 2:** Participant demographics (age, gender, ethnicity and SEP) for customer outlet surveys pre- and post-implementation

	Pre-implementation (*n* = 3,308)	Post-implementation (*n* = 3,270)
Age (*M* (s.d.))	41.0 (18.7)	40.4 (17.9)
Male	1,682 (51%)	1,527 (47%)
Female	1,622 (49%)	1,726 (53%)
SEP high	1,191 (36%)	1,585 (48%)
SEP low	2,117 (64%)	1,685 (52%)
White	2,787 (84%)	2,668 (82%)
Asian	208 (6%)	215 (7%)
Black	152 (5%)	126 (4%)
Mixed race	85 (3%)	212 (6%)
Other	56 (2%)	45 (1%)

‘SEP low’ indicates school leaving education qualifications or lower; ‘SEP high’ indicates education qualifications above school leaving or equivalent.

## Kcals purchased and consumed

At pre-implementation, a mean (*M*) of 1,007 kcals (standard deviation (s.d.) 630) and 909 kcal (s.d. 547) were purchased and consumed per customer, respectively. This was a smaller number of kcals compared with post-implementation (purchased *M* = 1,081 kcals, s.d. 650, consumed *M* = 983 kcals, s.d. 587). In adjusted models, there were no significant associations between time and kcals purchased (difference pre versus post, Beta (*B*) = 11.31, *P* = 0.564, 95% confidence interval (CI) −27.15 to 49.77)) or the number of kcals consumed (*B* = 18.51, *P* = 0.279, 95% CI −15.01 to 3,852.03) (Table 3). Bayes factors in unadjusted models demonstrated strong support for the null hypothesis for kcals purchased (BF^01^ of 546.51) and consumed (BF^01^ of 5,309.00). In supplementary models (Supplementary Information), there were no significant interactions between time and participant demographics ((1) SEP, (2) age, (3) gender and (4) ethnicity).

**Table 3:** Summary of regression models for kcals purchased, kcals consumed and kcal estimates

	Kcals purchased *B* (95% CI)	Kcals consumed *B* (95% CI)	Kcal estimates *B* (95% CI)
Post-implementation (versus pre-implementation)	11.31 (−27.15 to 49.77) *P* = 0.564	18.51 (−15.01 to 52.03) *P* = 0.279	61.21 (21.57 to 100.86) *P* = 0.002
Age (in years)	−1.18 (−2.14 to −0.22) *P* = 0.016	−0.87 (−1.76 to 0.01) *P* = 0.054	−1.10 (−2.09 to −0.12) *P* = 0.028
Male (versus female)	106.62 (76.76 to 136.48) *P* < 0.001	133.47 (105.80 to 161.15) *P* < 0.001	20.19 (−7.34 to 47.72) *P* = 0.151
Non-white (versus white)	−58.31 (−103.16 to −13.46) *P* = 0.011	−50.45 (−88.16 to −12.30) *P* = 0.010	−48.51 (−88.67 to −8.35) *P* = 0.018
Low SEP (versus high SEP)	−2.09 (−33.59 to 29.42) *P* = 0.897	10.07 (−17.71 to 37.86) *P* = 0.477	−107.71 (−146.75 to −68.67) *P* < 0.001
Midday (versus evening)	−156.01 (−222.99 to −89.04) *P* < 0.001	−114.25 (−169.27 to −59.22) *P* < 0.001	12.34 (−34.91 to 59.60) *P* = 0.609
Weekend (versus weekday)	99.59 (37.48 to 161.71) *P* < 0.001	73.62 (22.05 to 125.19) *P* = 0.005	37.86 (−12.08 to 87.80) *P* = 0.137
Entertainment (versus cafes)	66.18 (−42.19 to 174.55) *P* = 0.231	−70.68 (−165.83 to 24.47) *P* = 0.145	−20.79 (−107.89 to 66.31) *P* = 0.640
Fast food (versus cafes)	246.39 (177.22 to 315.57) *P* < 0.001	199.98 (146.30 to 253.66) *P* < 0.001	211.18 (163.72 to 258.63) *P* < 0.001
Pubs (versus cafes)	838.70 (774.26 to 903.15) *P* < 0.001	760.29 (705.04 to 815.53) *P* < 0.001	46.08 (−23.43 to 115.58) *P* = 0.194
Restaurants (versus cafes)	744.55 [(675.62 to 813.47) *P* < 0.001	662.93 (597.69 to 728.17) *P* < 0.001	300.15 (233.57 to 366.73) *P* < 0.001
IMD2 (versus IMD1)	−50.02 (−129.92 to 29.89) *P* = 0.220	−47.24 (−113.87 to 19.40) *P* = 0.165	−12.52 (−83.14 to 58.11) *P* = 0.728
IMD3 (versus IMD1)	−84.66 (−160.00 to −9.31) *P* = 0.028	−41.71 (−109.01 to 25.58) *P* = 0.224	8.05 (−49.44 to 65.54) *P* = 0.748
IMD4 (versus IMD1)	−71.81 (−148.32 to 4.71) *P* = 0.066	−53.83 (−119.60 to 11.93) *P* = 0.109	−38.85 (−106.43 to 28.73) *P* = 0.260
IMD5 (versus IMD1)	−70.53 (−147.05 to 5.99) *P* = 0.071	−46.47 (−109.95 to 17.00) *P* = 0.151	−66.77 (−121.98 to −11.55) *P* = 0.018
Kcals purchased	–	–	−0.64 (−0.69 to −0.59) *P* < 0.001
Number of observations	5,447	5,447	5,441
*R*^2^/*R*^2^ adjusted	0.378/0.377	0.380/0.379	0.361/0.359

Reference categories are in parentheses (for example, female and white). IMD1 represents the most deprived areas of England, and IMD5 represents the least deprived areas. In relation to kcal estimates, positive values represent an overestimation and negative values represent an underestimation of kcal content.

There were variations in the amount of kcal purchased and consumed based on participant demographics. Younger adults purchased more kcals than older adults (*B* = −1.18, *P* = 0.016, 95% CI −2.14 to −0.22), males purchased (*B* = 106.62, *P* < 0.001, 95% CI 76.76 to 136.48) and consumed (*B* = 133.47, *P* < 0.001, 95% CI 105.80 to 161.15) more than females and participants from a non-white ethnic background purchased (*B* = −58.31, *P* = 0.011, 95% CI −103.16 to −13.46) and consumed (*B* = −50.45, *P* = 0.010, 95% CI −88.16 to −12.30) less than those from a white ethnic background. Time of day (more kcals purchased for an evening meal) (*B* = 156.01, *P* < 0.001, 95% CI 222.99 to 89.04) and day of the week (more kcals purchased at weekends) (*B* = 99.59, *P* < 0.001, 95% CI 37.48 to 161.71) also was associated with the number of kcals purchased and consumed. Compared with cafes, people visiting pubs, restaurants and fast-food outlets purchased and consumed more kcals on average (Table 3).

## Participant kcal estimates

Across both timepoints, customers underestimated the number of kcals in their purchases. The amount of kcal underestimation reduced from pre- (247 kcals) to post-implementation (217 kcals) by 30 kcals in unadjusted analyses, with Bayes factors indicating support for the null hypothesis (BF^01^ of 8.76). Consistent with this in the adjusted models, kcal underestimation reduced post- versus pre-implementation (*B* = 61.21, *P* = 0.002, 95% CI 21.57 to 100.86) (Table 3). Age (younger adults underestimated less) (*B* = −1.10, *P* = 0.028, 95% CI −2.09 to −0.12), ethnicity (white participants underestimated less) (*B* = −48.51, *P* = 0.018, 95% CI −88.67 to −8.35) and SEP (high SEP underestimated less) (*B* = −107.71, *P* < 0.001, 95% CI −146.75 to −68.67) were associated with accuracy of kcal estimates. Compared with cafes, participants showed greater underestimation of kcal amounts from purchasing from restaurants (*B* = 300.15, *P* < 0.001, 95% CI 233.57 to 366.73) and fast-food outlets (*B* = 211.18, *P* < 0.001, 95% CI 163.72 to 258.63) (Table 3).

## Noticing and use of kcal labelling

In total, 16.5% (*n* = 402) of participants reported noticing kcal labelling pre-implementation whereas 31.8% (*n* = 959) reported noticing labelling post-implementation. Noticing kcal information was significantly higher post- versus pre-implementation (odds ratio 2.25, *P* < 0.001, 95% CI 1.84 to 2.73, BF^01^ <0.001, indicative of strong evidence against the null hypothesis) (Table 4). Of the people who reported noticing kcal labelling, 19% (*n* = 77) reported using kcal labelling to make their purchasing decision pre-implementation and 22% (*n* = 209) post-implementation. In the adjusted model, there was an association between time and reported use of kcal labelling (77/3,308 pre-implementation and 209/3,270 post-implementation) (odds ratio 2.15, *P* < 0.001, 95% CI 1.62 to 2.85) (Table 4). Of the people who reported using the kcal information, the majority of individuals, *n* = 249 (87%), reported using it to select lower-kcal options. For participant demographics, older adults noticed kcal labels more than younger adults but there was no significant difference in reported use. Gender and SEP influenced reported kcal noticing and use, with females reporting noticing and using kcal labels more than males and people from a high SEP reporting noticing and using labels more than those from a lower SEP. Participants were more likely to report noticing kcal labels when purchasing meals from a pub compared with a cafe, this may be due to variations in purchasing conditions such as displaying of kcal information and time spent viewing menus (for instance, in a cafe one often orders food and beverages together, whereas in a pub often one orders a drink first and then considers the food menu at a more leisurely pace). Participants were also more likely to report noticing kcal labels in outlets located in less affluent areas (IMD1) compared with more affluent areas (IMD5) (Table 4).

**Table 4:** Summary of regression models for reported noticing and use of kcal labelling

	Noticed kcal labels odds ratio (95% CI)	Used kcal labels odds ratio (95% CI)
Post-implementation (versus pre-implementation)	2.25 (1.84 to 2.73) *P* < 0.001	2.15 (1.62 to 2.85)* *P* < 0.001
Age (in years)	0.99 (0.99 to 0.99) *P* < 0.001	1.00 (1.00 to 1.01) *P* = 0.504
Male (versus female)	0.71 (0.62 to 0.81) *P* < 0.001	0.53 (0.41 to 0.69) *P* < 0.001
Non-white (versus white)	0.89 (0.73 to 1.09) *P* = 0.251	0.69 (0.47 to 1.01) *P* = 0.055
Low SEP (versus high SEP)	0.57 (0.49 to 0.66) *P* < 0.001	0.36 (0.27 to 0.47) *P* < 0.001
Midday (versus evening)	1.07 (0.87 to 1.32) *P* = 0.554	1.09 (0.76 to 1.55) *P* = 0.643
Weekend (versus weekday)	1.04 (0.83 to 1.30) *P* = 0.711	0.81 (0.54 to 1.21) *P* = 0.296
Entertainment (versus cafes)	0.38 (0.11 to 1.38) *P* = 0.089	0.24 (0.05 to 1.07) *P* = 0.027
Fast food (versus cafes)	1.04 (0.79 to 1.35) *P* = 0.797	0.77 (0.53 to 1.13) *P* = 0.176
Pubs (versus cafes)	1.81 (1.35 to 2.44) *P* < 0.001	0.90 (0.58 to 1.40) *P* = 0.631
Restaurants (versus cafes)	1.28 (0.94 to 1.75) *P* = 0.121	0.84 (0.52 to 1.36) *P* = 0.476
IMD2 (versus IMD1)	0.76 (0.56 to 1.03) *P* = 0.075	0.83 (0.53 to 1.31) *P* = 0.414
IMD3 (versus IMD1)	0.98 (0.74 to 1.30) *P* = 0.893	0.83 (0.53 to 1.27) *P* = 0.375
IMD4 (versus IMD1)	0.78 (0.58 to 1.06) *P* = 0.102	0.81 (0.49 to 1.33) *P* = 0.396
IMD5 (versus IMD1)	0.68 (0.51 to 0.89) *P* = 0.006	0.71 (0.45 to 1.11) *P* = 0.131
Number of observations	5,430	5,447
Pseudo *R*^2^	0.060	0.144

Reference categories in parentheses. IMD1 represents the most deprived areas of the United Kingdom, and IMD5 represents the least deprived areas.

## Discussion

### Statement of principal findings

The current study did not observe an association between kcals purchased or consumed in OHFS pre-implementation (2021) versus post-implementation (2022) of mandatory kcal labelling legislation in England (adjusted models). Additional analyses indicated that the lack of observed change did not differ on the basis of participant age, gender, ethnicity or SEP (education level). Reported noticing of kcal labelling post-implementation significantly increased, and customers more accurately estimated the kcal content of their purchases at post- versus pre-implementation. Despite this, there was only a small change in reported use of kcal labelling pre- versus post-implementation (77/3,308 pre-implementation and 209/3,270 post-implementation).

### Strengths and weaknesses of the study

This study examined purchasing, consumption and noticing and use of kcal labelling in the OHFS in England before versus after implementation of the national mandatory kcal labelling policy. This study recruited a large number of participants from a range of food outlets across multiple local authorities and area-level deprivation quintiles. Local authorities were purposively sampled to be generalizable across other areas of England and included outlets representing a large number of national chains.

A limitation of this study is the reliance on self-reporting of food purchased and consumed, which may introduce bias^32^. To mitigate inaccurate reporting, food purchases were recorded shortly after consumption and, where possible, customer receipts were used to verify purchases, although this was not always possible due to not being consistently issued by outlets. The calculation of kcals purchased was based on businesses’ reported kcal information for menu items. Previous research has indicated that this tends to be accurate but may be prone to underestimation of the energy content of some food items^33,34^; for example, one study found that kcal counts on menus were generally accurate, but restaurants underreported compared with fast food outlets^34^. We are not aware of any evidence suggesting that the accuracy of kcal information has changed over time, and so we presume this limitation is unlikely to introduce bias to the present results in relation to change estimates; however, kcal purchasing and consumption may be underestimated in this study. The use of objective verified measures of energy purchased and consumed would be preferable but was not feasible in this real-world policy evaluation. Further, it may be the case that people who were approached to take part in the study and declined may have purchased and consumed meals with a higher or lower energy content than participants sampled. Due to this, the data presented could be an underestimation or overestimation of the number of kcals purchased and consumed by people in the OHFS. However, there are no a priori reasons to expect this variance to be systematically different between pre- and post-policy implementation and, thus, not introducing substantial bias.

Although our study can conclude that the implementation of the policy was not associated with an immediate change in energy purchased and consumed, we cannot infer causality from a pre–post design owing to the inability to fully adjust for known and unknown confounders or compare data with any background trends (for example, pre-implementation data were collected shortly after coronavirus disease 2019 restrictions were removed in England).

Previous research has examined consumer behaviour changes following the implementation of kcal labelling in the OHFS; however, this has predominately been done in North America^35^. A small number of US studies have suggested that the introduction of kcal labelling was associated with small decreases in energy purchased in two fast food franchises and a supermarket chain selling prepared food^31,36,37^, but there has been no national evaluation of the US kcal labelling policy. In the United Kingdom, a limited number of trials in real-world settings have found no evidence that the introduction of kcal labelling reduced overall energy purchased^38,39^. Systematic reviews have produced similar findings, concluding that the quality of evidence is low and that kcal labelling has a small or no effect on the amount of energy selected, purchased and/or consumed^25–29^. The lack of an observed association between mandatory kcal labelling and energy purchased and consumed in the present study is not consistent with the three US studies described above^31,36,37^. However, these examined single fast food and supermarket chains (selling prepared food) in the United States, rather than the broad range of eligible OHFS businesses in the present study. In addition, contextual differences between the United States and England may also explain different findings, such as socio-demographic patterning, frequency of OHFS visits and/or food choice motives^14^.

Research has shown that a notable proportion of individuals do not notice kcal labels when eating out^29,40^. Larson et al.^40^ found that, out of 1,830 US adults, only 52.7% were aware of kcal labelling when eating at a restaurant in the past month, with 38.2%, among those who noticed labelling reporting that they did not use it when making their purchase decision. In our study, only around 30% of people post-implementation reported noticing kcal labelling. Of those people, only 22% (209/3,270 across all participants post-implementation) reported that they used this information when making their purchasing decisions. Despite a small increase (3%) in reported usage post-implementation, this may explain the lack of an association with consumer purchasing found in this study. Although there was an increase in participants who reported noticing kcal labelling following mandatory implementation (an increase from 17% to 32%), these figures are still relatively low compared with figures from the United States (for example, 60% noticed kcal labelling)^41^. Labelling guidance is similar between the United States^42^ and England. However, a US study examining compliance found that 94% of 197 chains had implemented kcal labelling post-regulations^43^, which is higher than compliance rates found in the England (80%)^44^. This greater level of compliance may have contributed to higher reported noticing and use of kcal labels in the United States and may have contributed to lower levels of reported noticing and use of kcal labels in this study. The lower compliance rates found in England^44^ has potentially limited the effectiveness and impact of the policy on customer noticing and use of kcal labels and, in turn, probably impacts on kcals purchased and consumed.

When making dietary choices, individuals of lower SEP are more likely to report being less motivated by weight management or the healthiness of food^14^. In our study, people from a lower SEP demonstrated greater underestimation of the energy content of meals purchased and lower reported noticing and use of kcal labelling in individuals from lower SEP. However, there was no evidence that the change in kcals purchased or consumed pre- versus post-implementation differed on the basis of any demographics, including SEP. This is consistent with previous systematic review evidence indicating that the effect of kcal labelling on consumer purchasing and consumption does not differ on the basis of SEP^45^. It should be noted that, in the current study, SEP was characterized only on the basis of highest education level, and although most appropriate for this study, education level does not consider factors such as generational differences in education opportunities or financial resources^46,47^.

The kcal content of OHFS meals purchased and consumed in this study was high compared with UK public health recommendations of 600 kcals per meal^48^. This finding is broadly consistent with previous research that food purchased in the OHFS is high in kcals^1,49^. Previous research has indicated socio-demographic differences in purchasing and consumption in the OHFS^50^, and this study observed some of these variations. Consistent with previous research, it was found that males purchased and consumed more than females^51^, younger adults purchased and consumed more than older age groups^52,53^, and there were ethnicity variations in purchasing and consumption, with participants from a white ethnic background purchasing and consuming more than those from a non-white ethnic background^54^.

There were no observed changes in customer purchasing or consumption of energy in the OHFS following the implementation of mandatory kcal labelling in England in the current study. These findings indicate that the current implementation of mandatory kcal labelling legislation is unlikely alone to have substantial impacts on out-of-home eating. However, it may be the case that alongside other policies it may contribute to wider and more substantial impacts on diet and public health. The requirement to provide kcal labelling on OHFS menus in England was introduced alongside other public health policies, such as a tax on sugary soft drinks, restrictions on advertising unhealthy foods and increased funding for physical activity in schools^55^. The kcal labelling policy alone may not have large impacts on OHFS consumer purchasing, but instead contributes to improved wider public health alongside other policies, especially through the gradual shift of social norms. Future research would benefit from examining the impacts of multiple newly implemented policies and the combined impact of these policies on public health.

Mandatory kcal labelling in the OHFS could lead to a reduction in kcal consumption through two pathways: by influencing individuals’ food choices and through menu reformulation^24^. The current study did not find a reduction in kcals due to consumer behaviour change. However, the policy may have impacted overall kcal consumption via menu reformulation. A recent study^56^ examined changes in online menu information from large out-of-home food outlets in England between September 2021 and September 2022, finding a small reduction in mean kcals after the implementation of the kcal labelling policy. This reduction was driven by the removal of higher-kcal menu items and the introduction of lower-kcal menu items. The study found no changes pre- and post-implementation of the policy in kcal content for continuously available items. This indicates that if reductions in population level kcal consumption following the policy have occurred, they may have been more likely to have been driven by menu reformulation rather than consumer behaviour change.

Complementary research examining kcal labelling legislation compliance in OHFS outlets in England pre- versus post-implementation found that, while the provision of labelling increased pre–post, only 80% of sampled OHFS provided kcal labelling post-regulations^44^. When examining the quality of this labelling it was found that only 15% of outlets met all kcal labelling compliance criteria post-regulations, with a minority of outlets not presenting kcal labelling in a clear (33%) or legible (29%) manner^44^. A lack of compliance with labelling legislation in outlets may therefore have contributed to the lack of change in energy purchased and consumed observed in the present study. Enforcement of the policy is currently being conducted at a local authority level, with local authorities encouraged to attempt to improve compliance with the food business before issuing a £2,500 fine^23^. Greater and stricter enforcement of labelling legislation may be required to improve compliance and increase the likelihood that consumers notice and use kcal labelling in the OHFS, which in turn may lead to impacts on customer purchasing and consumption.

A potential barrier to the use of kcal labelling in the OHFS may be a lack of public understanding of the kcal information presented. Research conducted in the United States found that only 64–73% of the general public was able to accurately report daily kcal needs^57^. Increased awareness and availability of kcal labelling may have helped to improve the public’s knowledge of the kcal content of foods from the OHFS, and this was reflected by more accurate customer kcal estimates post-implementation found in this study. However, public education campaigns about kcal requirements may be required to further increase understanding and, in turn, may increase usage of kcal labelling in the OHFS. Alternatively, additional labelling formats that provide more context and/or define foods as ‘low’, ‘moderate’ or ‘high’ kcals may aid understanding^58^. These types of nutrition label have shown to be effective on the front of package labels aiming to improve people’s judgements of healthiness of food items^59^, with traffic light labels leading to the greatest accuracy at identifying healthier food items compared with other labelling types^60^. Labelling types that provide greater content and guidance may aid understanding and may increase the likelihood of customers selecting healthier food items in OHFS contexts. Future research is required to fully examine the extent of the UK public’s understanding of kcal labelling in the OHFS and if education campaigns and other labelling formats have the potential to promote greater use of kcal information.

As this study included only education as an indicator of SEP, future research would benefit from examining the effects of kcal labelling on consumer behaviour and whether this differs on the basis of multiple indicators of SEP, such as household income.

## Conclusions

The current study did not observe a significant decrease in the number of kcals purchased or consumed in OHFS outlets following the introduction of mandatory kcal labelling policy in England. A lack of compliance with labelling legislation found in previous research^44^ may have contributed to the lack of change in energy purchased and consumed observed in the present study.

## Methods

This research complies with all relevant ethical regulations, and ethical approval was granted by the University of Liverpool’s Ethics Committee (project ID 10137). All participants provided informed verbal consent, and participants were offered a £5 shopping voucher for taking part in the study. The study protocol and analysis strategy were pre-registered on Open Science Framework (https://osf.io/pfnm6/).

### Study design

We used a pre-implementation (August to December 2021) versus post-implementation (August to December 2022) observational study design in which we visited OHFS outlets and surveyed customers in four areas of England before and after the introduction date of the mandatory kcal labelling legislation on 6 April 2022^23^.

### Outlet sampling procedure

Four local authorities in England were purposively selected for sampling to ensure representation across quintiles of deprivation (assessed using the Index of Multiple Deprivation (IMD)^61^ at the local authority level) and geographical coverage across the South, North, Midlands and London areas of England. The four local authorities sampled were Liverpool (IMD1 northern region), Dudley (IMD2 midlands), Milton Keynes (IMD3, IMD4 South) and Richmond upon Thames (IMD4, IMD5 London). IMD1 reflects the most deprived areas and IMD5 the least deprived areas defined at the lower layer super output area (LSOA) to better capture small area geographic variations in IMD. Businesses subject to the mandatory kcal labelling policy were identified using the Inter-Departmental Business Register^62^. This is a list of UK businesses and their core characteristics, including principal activities and the number of employees, used by the government for statistical purposes with the principal activities of businesses defined using Standard Industrial Classification codes. Codes likely to include businesses serving food were identified (see Supplementary Section 1 for the full list of Standard Industrial Classification codes used), and then those that were not large businesses with >250 employees globally were excluded. Within the four local authorities, individual outlets belonging to each identified large business (individual businesses could contribute to multiple outlets, for example, chain restaurants) were identified using Ordnance Survey Points of Interest data from September 2020^63^. Following this, we used stratified random sampling by business type and IMD quintile within each local authority to select outlets for inclusion. Business types were categorized by Ordnance Survey as follows: restaurants; pubs and bars; retail; hotels; cafes; fast food; attractions; and entertainment. Outlets that were found to be closed, not selling food subject to the mandatory kcal labelling policy, or would not permit data collection on visiting at the pre-implementation assessment were replaced by resampling.

### Customer exit survey sampling procedure

Exit surveys with customers from sampled outlets were conducted to measure the number of kcals purchased and consumed, kcal knowledge of meal purchases, and self-reported noticing and use of kcal labelling. To be eligible for inclusion, participants were required to have purchased at least one food item from the selected outlet and be aged 16 years or over.

Researchers stood outside the selected food outlets during peak operating times (typically 12:00–21:00, Wednesday to Sunday) and approached all customers as they entered or exited the outlet. Where possible, data collection timepoints (weekend or weekday and evening or midday) were kept consistent across the pre and post data collection periods. Participants completed a short exit survey lasting approximately 5–10 min per participant (survey questions in Supplementary Section 2). Participants were initially told that the study was investigating dining habits to minimize influencing participants’ purchasing behaviour and avoid increasing their focus on kcal labelling. Participants were later debriefed with a full explanation of the study’s aims. Basic demographic information was collected (age, gender, ethnicity and highest education level), with education level used to indicate participants’ SEP (lower SEP: school level qualifications or lower; higher SEP: post-school level qualifications). Participants were asked to estimate the total number of kcals in their purchases. Following this, participants were asked about whether they noticed kcal labelling provided by the outlet (yes/no), whether they used this when making their purchases (yes/no) and, if yes, why (to select lower-kcal options, to select higher-kcal options, other) and how (selected alternative meal option, selected a smaller or larger portion, made a meal substitution or customization). Participants were then asked to report the food and drink items that they purchased from the outlet for their own consumption and to estimate any food that was shared or was not consumed. Self-reporting of shared items and leftovers was used to calculate consumption values for each participant. Whenever possible, customers were asked to provide a receipt to verify purchases; however, many outlets were not issuing receipts during data collection owing to hygiene concerns and procedural changes related to the coronavirus disease 2019 pandemic. Data were collected on the availability and quality of kcal labelling in a subset of food outlets at both pre and post timepoints, with data reported in a recent publication^44^. The proportion of outlets providing kcal labelling at any point-of-choice increased from pre (21%) to post (80%) policy implementation.

### Sample size

The sample size required for customer exit surveys was based on results from a Cochrane review that included 28 studies examining the effect of nutritional labelling on purchasing and consumption^30^. The sample size was calculated to detect a 47 kcal reduction from a baseline mean of 706 kcal (s.d. 326) purchased per individual (7% reduction) as reported in the Cochrane review. Assuming a modest intra-class correlation of kcals purchased within outlets of 0.39 (ref. ^64^) and 10 participants per outlet, we estimated required sample sizes of *N* = 3,440 at pre and post from 344 outlets to detect a 7% reduction in energy purchased or consumed per participant with 80% power at *α* = 0.05.

Permission from outlets was withheld for data collection in retail, attraction and hotel outlets, resulting in 21 outlets from these categories being excluded from data collection. The sample for pre-implementation consisted of 330 outlets (from 76 unique businesses) including cafes, fast food, pubs, restaurants and entertainment, resulting in a pre-implementation participant sample size of *n* = 3,308 (approximately 10 people per outlet, with some variations when participants were recruited in groups). At the post-implementation observation, we attempted to conduct surveys at the same outlets sampled pre-implementation. Due to the closure of some of the outlets, five outlets were excluded from the sample (Fig. 1), resulting in a post-implementation participant sample of *n* = 3270.

### Estimation of meal kcal content

The kcal content of each participant’s food and drink purchases was estimated using information from MenuTracker^65^. MenuTracker is a database of web-scraped nutritional information on menu items in large UK OHFS businesses. Data are collected quarterly, and data from September 2021 were used to calculate kcal content for the pre-implementation data collection and September 2022 for the post-implementation data collection to minimize the effects of seasonal variation on menu items available. Nutritional content was sought from the business’s websites in instances where the kcal content was not available from MenuTracker. In instances where multiple menu item options were available in the database, or the item was not identifiable (for example, if it was unclear which menu item was purchased from the participant’s description), the closest matching item (or mean of items) was used or the item was coded as missing (Supplementary Section 3).

### Data exclusions

If the total number of kcals the participant purchased was unavailable or incomplete, they were excluded from the kcal purchased, kcal consumed and kcal estimates primary analyses. However, these participants were retained for the analyses of noticing and use of kcal labelling. The number of exclusions is reported by reason of missing data in Supplementary Section 3.

### Data analysis

To examine whether outcome variables differed pre- versus post-implementation, linear and logistic regression were used with time (pre-implementation/post-implementation), age, gender, ethnicity and SEP as demographic adjustment variables and outlet type and outlet location IMD (at the LSOA level, calculated from outlet postcode) as outlet adjustment variables, and with robust standard errors to account for clustering by outlet. Time of day (lunch versus dinner) and day (weekday versus weekend) were included as covariates. Analyses were conducted using the ‘estimatr’^66^ and ‘clubSandwich’^67^ packages in R version 4.3.1. Outcome variables were number of kcals purchased; number of kcals consumed (adjusting for leftover and items shared estimates); accuracy of customer kcal estimates (customer estimate minus actual kcal amount determined via MenuTracker); and kcal noticing and use (both yes versus no). As meal kcal content could influence the accuracy of customer kcal estimates^68^, models examining the accuracy of kcal estimates additionally included total kcals purchased. We planned to use local authority as a further variable in models, but this was highly collinear with outlet IMD so it was removed. However, in supplementary analyses (Supplementary Section 5), we replaced IMD with local authority in the models and the results were consistent. When analysing the use of kcal labels, missing values (this question was not asked if participants did not report noticing kcal labels) were coded as ‘did not use’. An *α* value of 0.05 for statistical significance was used for the main analyses described above. Additional models for kcals purchased and consumed were examined if the effect of time (pre versus post) was moderated by participant demographics by adding interaction terms between time and (1) SEP, (2) age, (3) gender and (4) ethnicity. For these additional analyses including interactions, an *α* value of 0.01 (99% confidence intervals) was used to determine statistical significance to account for the relatively large number of additional analyses conducted. Bayes factors were computed for unadjusted and unclustered simple models (the association between time and outcomes of kcals purchased and consumed, kcal estimation accuracy and noticing of labels) using the ‘ttestBF’ and ‘contingencyTableBF’ functions from the ‘bayesfactor’ package in R. We tested one-sided hypotheses that kcals purchased and consumed would be lower at post-implementation. We report BF^01^, in which a value >1 is indicative of support for the null model (absence of evidence for change) over the alternative model (a reduction in kcal purchasing or consumption as post-implementation). The analysis protocol was registered on the Open Science Framework (https://osf.io/pfnm6/) with minor deviations made from the registered protocol reported in Supplementary Section 4. The lead author affirms that this manuscript is an honest, accurate and transparent account of the study being reported; that no important aspects of the study have been omitted; and that any discrepancies from the study as planned have been explained.

### Reporting summary

Further information on research design is available in the Nature Portfolio Reporting Summary linked to this article.

## Supplementary information

Supplementary Information Supplementary Tables and Information.

Reporting Summary

## Data Availability

Data from this study are available on the Open Science Framework at https://osf.io/rva8g/.

## Code availability

Analysis code from this study is available on the Open Science Framework at https://osf.io/rva8g/.
