# Supplementary material for: Evaluating the association between the introduction of mandatory calorie labelling and energy consumed using observational data from the out-of-home food sector in England
Source: Nat Hum Behav. 2024 Nov 25;9(2):277–86. doi: 10.1038/s41562-024-02032-1 (PMC11860223; doi:10.1038/s41562-024-02032-1)
Supplement: Supplementary file 1 — Supplementary Tables and Information. [file 41562_2024_2032_MOESM1_ESM.pdf]

nature human behaviour

Supplementary information https://doi.org/10.1038/s41562-024-02032-1

Evaluating the association between the introduction of mandatory calorie labelling and energy consumed using observational data from the out-of-home food sector in England

In the format provided by the authors and unedited

Supplementary Materials

Table of contents **Page Number**

1. Section 1: List of Standard Industrial Classification codes used for sampling 2
2. Section 2: List of survey questions 3
3. Section 3: Missing participant data and missing outlets reported by reason 4
4. Section 4: Report of data analysis deviations from registered analysis protocol 5
5. Section 5: Supplementary analyses with LA rather than IMD in file 6
6. Section 6: Results with multiple imputation methods to impute the missing kcal information data 7
7. Section 7: Supplementary analyses with interactions with participant demographics 8

Section 1: List of Standard Industrial Classification codes used for sampling

Large businesses with the following Standard Industrial Classification (SIC) codes (and hence in the following IDBR sections) that are subject to the policy and thus included in this study:

Section I (accommodation and food service activities)

Within section G:

- SIC 47.11- Retail sale in non-specialised stores with food, beverages or tobacco predominating.
- SIC 47.24- Retail sale of bread, cakes, flour confectionery and sugar confectionery in specialised stores.
- SIC 47.29 Other retail sales of food in specialised stores.

Within section R:

- SIC 91.03-Operation of historical sites and buildings and similar visitor attractions
- SIC 91.04-Botanical and zoological gardens and nature reserve
- SIC 93.11-Operation of sports facilities
- SIC 93.12-Activities of sports clubs
- SIC 93.13-Fitness facilities
- SIC 93.21-Activities of amusement parks and theme parks

Within section J:

- SIC 59.14-Motion picture projection activities

Section 2: List of survey questions (response options)

1. What is your age?
2. What gender do you identify as? (Male/ Female/ other)
3. What is your ethnicity?
4. What is the highest degree or level of school you have completed?
5. Can you provide an estimate of the total number of kcals in the food and drink that you purchased for your own consumption?
6. Did you notice the use of kcal labelling in the food outlet? (Yes/No)
7. Did you use the provided kcal labelling when purchasing food and drink? (Yes/No)
8. Why did you use kcal labelling when making your purchases? (To select lower kcal options/ To select higher kcal options/ Other)
9. How did you use kcal labelling to select lower/higher kcal options? (Selected a smaller or larger portion size/ Made a customisation or meal substitution/ Altered choice of food or drink/ Other)
10. What food and drink items did you purchase for your own consumption?
11. Did you make any meal substitutions or customisations? e.g. salad instead of chips? (Yes/No)
12. What customisations/substitutions were made?
13. Did you share any of the food or drink items? (Yes/No)
14. What items were shared?
15. Were any food or drink items leftover and not fully consumed? (Yes/No)
16. What food or drink items were leftover and not fully consumed?
17. What percentage of the item was leftover and not consumed? Please provide an estimate for each individual food or drink item that was leftover.

Section 3: Missing participant data and missing outlets reported by reason

Table 1. Pre-implementation number of participants removed from purchasing, consumption and accuracy of kcal content analyses due to missing data, split by outlet type.

	**Total Participants (N=856)**	**Cafes (N= 39)**	**Pubs (N= 377)**	**Fast-food (N= 27)**	**Restaurants (N= 378)**	**Entertainment venues (N= 35)**
**Nutritional data not available from outlet**	802	27	376	9	355	35
**Food items not identifiable**	54	12	1	18	23	0

Note: Food items not identifiable were items unable to be matched from participant description to food items listed on the outlet's menu

Table 2. Post-implementation number of participants removed from purchasing, consumption and accuracy of kcal content analyses due to missing data split by outlet type.

	**Total Participants (N=255)**	**Cafes (N=25)**	**Pubs (N=108)**	**Fast-food (N=15)**	**Restaurants (N=83)**	**Entertainment venues (N=24)**
**Nutritional data not available from outlet**	219	21	99	11	68	20
**Food items not identifiable**	36	4	9	4	15	4

Note: Food items not identifiable were items unable to be matched from participant description to food items listed on the outlet's menu

In total, 856 participants (25.8%) had missing data for analyses examining kcal purchased, consumed and knowledge of kcal content pre-implementation and 255 participants (7.80%) post-implementation.

Section 4: Report of data analysis deviations from registered analysis protocol

Additional models for kcals purchased and consumed were run to examine if the effect of time (pre vs. post) was moderated by participant demographics by adding interaction terms between time and (1) SEP, (2) age, (3) gender, (4) ethnicity. For these additional analyses including interactions, 99% confidence intervals are reported and the p-value for statistical significance was set at .01. We used a more stringent p-value as these analyses were exploratory.

To address missing data, we originally planned to run multiple analysis models first including only the outlets that we had kilocalorie information available at pre and post data collection and a second model including outlets in which kilocalorie information was linked retrospectively and not initially available at baseline. However, due to changes in menu and food item reformulation being likely to occur during these time points we instead opted to address the missing data using multiple imputation methods to impute the missing kcal information data. We report both models, one without the use of multiple imputation methods in the main manuscript and the other including multiple imputation methods in the supplementary materials for kcal purchased, consumed and kcal estimates.

Section 5: Supplementary analyses with Local Authority rather than IMD

Table 3. Inclusion of Local Authority (rather than IMD) in the confirmatory models.

	Kcals Purchased<br>B [95% CI]	Kcals Consumed<br>B [95% CI]	Kcal Estimates<br>B [95% CI]
Post-implementation (v. Pre-implementation)	11.72 [-26.73, 50.16]	19.92 [-13.57, 53.41]	60.46 [20.84, 100.09]*
Age	-1.33 [-2.25, -0.41]*	-1.02 [-1.87, -0.17]*	-1.20 [-2.16, -0.23]*
Male (v. Female)	106.72 [77.31, 136.13]*	134.66 [107.19, 162.13]*	19.94 [-7.25, 47.41]
Non-White (v. White)	-64.72 [-109.43, -20.00]*	-56.89 [-94.89, -18.90]*	-48.74 [-89.10, -8.38]*
Low SEP (v. high SEP)	-5.77 [-37.36, 25.83]	2.94 [-24.40, 30.27]	-100.43 [-139.50, -61.36]*
Midday (v. Evening)	-162.44 [-228.82, -96.07]*	-116.93 [-170.94, -62.92]*	7.73 [-39.28, 54.73]
Weekend (v. Weekday)	87.36 [25.75, 148.97]*	61.05 [10.22, 111.87]*	24.36 [-26.70, 75.42]
Entertainment (v. Cafes)	74.82 [-35.64, 185.27]	-58.01 [-147.76, 31.74]	- 13.33 [-108.95, 82.30]
Fast food (v. Cafes)	241.23 [170.45, 312.01]*	191.88 [136.73, 247.03]*	214.54 [164.98, 264.11]*
Pubs (v. Cafes)	828.52 [760.80, 896.25]*	750.85 [694.76, 806.93]*	43.71 [-26.55, 113.97]
Restaurants (v. Cafes)	727.64 [659.15, 796.14]*	650.56 [585.57, 715.56]*	-288.32 [221.05, 355.59]*
Liverpool (v. Dudley)	-71.29 [-141.66, -0.91]*	-85.58 [-145.68 – -25.48]*	29.23 [-28.72, 87.18]
Milton Keynes (v. Dudley)	41.6 [-36.52, 119.72]	24.31 [-37.98, 86.61]	60.38 [2.09, 118.68]*
Richmond (v. Dudley)	-53.91 [-133.31, 25.48]	-69.05 [-133.51, -4.59]*	-2.69 [-57.75, 52.36]
Kcals Purchased	-	-	-0.64 [-0.69, -0.59]*
Num. obs.	5447	5447	5441
R^2 / R^2 adjusted	0.380/ 0.379	0.385 / 0.384	0.360/ 0.359

Table legend: Reference categories are in brackets (e.g. Female, White). SEP= Socioeconomic position.
** Significant at p < .05 level*

Section 6: Results with multiple imputation methods to impute the missing kcal information data

We conducted multiple imputation on the data set, given that we were unable to obtain outcome data for kcals purchased if nutritional information was not available. We limited imputation analyses to kcals purchased as in main analyses kcals purchased and consumed results were the same. There were also small amounts of missing data for other exposure variables (<1%). Overall, 1,112 values were missing, with 857 missing pre-implementation and 255 missing post-implementation.

To conduct multiple imputation, we used the 'mice' (multiple imputation using chained equations) package in R. We created 20 imputations, in line with recommendations (Austin et al, 2021). Note, we interpret these findings with caution as there is some debate as to whether to include outcome variables (Van Ginkel et al, 2020). To impute our variables, we included the following information into our multiple imputation: outlet ID, local authority, outlet type, IMD, day (weekday vs weekend), time (lunch vs dinner), age, gender, ethnicity, SEP, estimated kcal consumed, label noticed, and implementation (pre- vs post-). The results when including imputed data were largely similar. Notably, the effect of the policy implementation remained non-significant.

Table 4. Results including imputed data for predictors of kcals purchased

	Estimate	99% CIs
Post-implementation (v. Pre-implementation)	15.01	-12.36, 42.38
Age	-1.20	-1.94, -0.46
Male (v. Female)	106.73	78.73, 134.73*
Non-White (v. White)	-62.97	-96.95, -28.99*
Low SEP (v. high SEP)	5.29	-23.91, 34.49
Midday (v. Evening)	-162.58	-198.12, -127.05*
Weekend (v. Weekday)	98.22	63.49, 132.95*
Entertainment (v. Cafes)	47.25	-39.11, 133.62
Fast food (v. Cafes)	241.71	213.34, 270.09*
Pubs (v. Cafes)	828.53	788.03, 869.02*
Restaurants (v. Cafes)	742.60	702.93, 782.28*
IMD2 (v. IMD1)	-59.66	-100.27, -19.04*
IMD3 (v. IMD1)	-84.94	-125.06, -44.83*
IMD4 (v. IMD1)	-76.37	-120.65, -32.1*
IMD5 (v. IMD1)	-69.91	-112.76, -27.05*

Table legend: Reference categories in brackets. IMD= Indices of Multiple Deprivation (location of outlet), SEP= Socioeconomic position. IMD1 represents the most deprived areas of the UK and IMD5 represents the least deprived areas.
** Significant at p < .01 level*

Section 7: Supplementary analyses with interactions with participant demographics.

We ran additional models for kcals purchased and consumed but added interaction terms between time and (1) SEP, (2) Age, (3) Gender, (4) ethnicity to examine whether the effects of kcal labels were equivocal across demographic groups. None of these interactions were statistically significant at p <.01.

Table 5. Additional model for kcal purchased and consumed with added interaction terms.

	Kcals Purchased B [99% CI]	Kcals Consumed B [99% CI]
Post-implementation (v. Pre-implementation)	-51.19 [-171.87, 69.48]	-11.79 [-121.47, 97.89]
Age	-1.81 [-3.41, -0.22]*	-1.38 [-2.85, 0.10]
Male (v. Female)	74.67 [20.06 , 129.27]*	111.30 [61.07, 161.53]*
Non-White (v. White)	-74.74 [-152.58, 3.10]	-55.50 [-127.24, 16.24]
Low SEP (v. high SEP)	19.08 [-38.27, 76.44]	41.29 [-10.79, 93.38]
Midday (v. Evening)	-156.59 [-244.27, -68.90]*	-115.13 [-187.04, -43.22]*
Weekend (v. Weekday)	99.59 [18.02, 181.15]*	73.56 [6.03, 141.08]*
Entertainment (v. Cafes)	65.56 [-78.58, 209.69]	-70.58 [-194.64, 53.48]
Fast food (v. Cafes)	247.15 [156.09, 338.20]*	200.82 [130.17, 271.46]*
Pubs (v. Cafes)	837.37 [753.18, 921.55]*	758.94 [686.88, 831.00]*
Restaurants (v. Cafes)	743.49 [652.81, 834.17]*	661.97 [576.35, 747.58]*
IMD2 (v. IMD1)	-50.16 [-155.00, 54.68]	-47.32 [-134.58, 39.93]
IMD3 (v. IMD1)	-83.20 [-182.12, 15.71]	-41.08 [-129.47, 47.30]
IMD4 (v. IMD1)	-70.74 [-171.03, 29.55]	-52.60 [-138.66, 33.46]
IMD5 (v. IMD1)	-71.42 [-171.95, 29.10]	-47.41 [-130.68, 35.86]
Time * Ethnicity	29.74 [-69.37, 128.85]	10.11 [-78.23, 98.44]
Time * Age	1.27 [-1.11, 3.64]	1.02 [-1.19, 3.22]
Time * SES	-38.15 [-114.28, 37.99]	10.11 [-126.30, 14.62]
Time * Gender	57.92 [-13.25, 129.09]	39.93 [-25.28, 105.14]
N	5447	5447
R2 / R2 adjusted	0.38 /0.38	0.38 /0.38

Table legend: Reference categories in brackets. IMD= Indices of Multiple Deprivation (location of outlet), SEP= Socioeconomic position. IMD1 represents the most deprived areas of the UK and IMD5 represents the least deprived areas.
** Significant at p < .01 level*
