# Supplementary material for: Evaluating the association between the introduction of mandatory calorie labelling and energy consumed using observational data from the out-of-home food sector in England
Source: Nat Hum Behav. 2024 Nov 25;9(2):277–86. doi: 10.1038/s41562-024-02032-1 (PMC11860223; doi:10.1038/s41562-024-02032-1)
Supplement: Supplementary file 2 — Reporting Summary [file 41562_2024_2032_MOESM2_ESM.pdf]

nature portfolio

Corresponding author(s): Megan Polden

Last updated by author(s): 19.09.2024

Reporting Summary

Nature Portfolio wishes to improve the reproducibility of the work that we publish. This form provides structure for consistency and transparency in reporting. For further information on Nature Portfolio policies, see our Editorial Policies and the Editorial Policy Checklist.

Statistics

For all statistical analyses, confirm that the following items are present in the figure legend, table legend, main text, or Methods section.

n/a	Confirmed	
☐	☒	The exact sample size (*n*) for each experimental group/condition, given as a discrete number and unit of measurement
☒	☐	A statement on whether measurements were taken from distinct samples or whether the same sample was measured repeatedly
☐	☒	The statistical test(s) used AND whether they are one- or two-sided<br>*Only common tests should be described solely by name; describe more complex techniques in the Methods section.*
☐	☒	A description of all covariates tested
☐	☒	A description of any assumptions or corrections, such as tests of normality and adjustment for multiple comparisons
☐	☒	A full description of the statistical parameters including central tendency (e.g. means) or other basic estimates (e.g. regression coefficient) AND variation (e.g. standard deviation) or associated estimates of uncertainty (e.g. confidence intervals)
☐	☒	For null hypothesis testing, the test statistic (e.g. *F*, *t*, *r*) with confidence intervals, effect sizes, degrees of freedom and *P* value noted<br>*Give P values as exact values whenever suitable.*
☐	☒	For Bayesian analysis, information on the choice of priors and Markov chain Monte Carlo settings
☐	☒	For hierarchical and complex designs, identification of the appropriate level for tests and full reporting of outcomes
☐	☒	Estimates of effect sizes (e.g. Cohen's *d*, Pearson's *r*), indicating how they were calculated

Our web collection on statistics for biologists contains articles on many of the points above.

Software and code

Policy information about availability of computer code

Data collection	No custom code was used during data collection
Data analysis	Analyses were conducted using the 'estimatr' and 'clubSandwich' packages in R version 4.3.1. The data and analysis code are available here (https://osf.io/rva8g/).

For manuscripts utilizing custom algorithms or software that are central to the research but not yet described in published literature, software must be made available to editors and reviewers. We strongly encourage code deposition in a community repository (e.g. GitHub). See the Nature Portfolio guidelines for submitting code & software for further information.

Data

Policy information about availability of data

All manuscripts must include a data availability statement. This statement should provide the following information, where applicable:

- Accession codes, unique identifiers, or web links for publicly available datasets
- A description of any restrictions on data availability
- For clinical datasets or third party data, please ensure that the statement adheres to our policy

Data and analysis code from this study is available on the Open Science Framework (https://osf.io/rva8g/).

Research involving human participants, their data, or biological material

Policy information about studies with human participants or human data. See also policy information about sex, gender (identity/presentation), and sexual orientation and race, ethnicity and racism.

Reporting on sex and gender	N=6578 participants were recruited, n=3308 pre-implementation and n=3270 post-implementation. Across both time points, recruited participants were of a similar mean age and a comparable distribution of gender and ethnicity. 51% od the sample were male at the post-implementation time point and 47% were male at the post-implementation time point.
Reporting on race, ethnicity, or other socially relevant groupings	We collected data on education level which was used as an indicator of socioeconomic status. Information was collected on the participant's ethnicity and analysis was conducted to examined if purchasing and consumption of OHFS food was influenced by socioeconomic status, gender, age and ethnicity. Participant information was self-reported.
Population characteristics	N=6578 participants were recruited, n=3308 pre-implementation and n=3270 post-implementation. Across both time points, recruited participants were of a similar mean age (41 years old pre-implementation, 40.4 years old post-implementation) and a comparable distribution of gender and ethnicity (84% white pre-implementation and 82% white post-implementation). It should be noted that there was a higher proportion of lower SEP participants in the pre-implementation (64% sample compared to post-implementation (52%). 51% of the sample were male at the pre-implementation time point and 47% were male at the post-implementation time point.
Recruitment	Participants were recruited via opportunity sampling. Researchers stood outside the selected food outlets during peak operating times (typically 12 pm – 9 pm, Wednesday to Sunday) and recruited customers as they entered or exited the outlet. Participants completed a short exit survey. Ethics oversight Ethical approval was granted by the University of Liverpool. It should be noted that self-selection bias may have influenced the result of this study. People who decided to take part in the study may have been more health motivated which may have influenced the results. It is possible that self-selection bias lead to the sample only being representative of part of the population.
Ethics oversight	Ethical approval was granted by the University of Liverpool's Ethics Committee (Project ID: 10137)

Note that full information on the approval of the study protocol must also be provided in the manuscript.

Field-specific reporting

Please select the one below that is the best fit for your research. If you are not sure, read the appropriate sections before making your selection.

☐ Life sciences ☒ Behavioural & social sciences ☐ Ecological, evolutionary & environmental sciences

For a reference copy of the document with all sections, see nature.com/documents/nr-reporting-summary-flat.pdf

Behavioural & social sciences study design

All studies must disclose on these points even when the disclosure is negative.

Study description	This study was a real-world observational quantitative study. We examined whether the implementation of mandatory kilocalorie (kcal) labelling policy in England was associated with a change in consumer behaviour. Researchers visited the same large out-of-home food sector outlets subject to kcal labelling legislation pre and post-implementation and conducted customer exit surveys with 6578 customers from 330 outlets. Kcals purchased and consumed by customers, knowledge of purchased kcals, and reported noticing and use of kcal labelling were examined.
Research sample	Participants were recruited via opportunity sampling and we people aged over 16 years old visiting large food outlet chains located in England. Four local authorities in England were purposively selected for sampling to ensure representation across quintiles of deprivation (assessed using the Index of Multiple Deprivation (IMD) at the local authority level) and geographical coverage across the South, North, Midlands and London areas of England. The four local authorities sampled were Liverpool (IMD1 northern region), Dudley (IMD2 midlands), Milton Keynes (IMD3, South) and Richmond upon Thames (IMD5 London). Businesses subject to the mandatory kcal labelling policy were identified using the Inter-Departmental Business Register. This is a list of UK businesses and their core characteristics, including principal activities and the number of employees, used by the government for statistical purposes with the principal activities of businesses defined using Standard Industrial Classification codes. Codes likely to include businesses serving food were identified and then those that were not large businesses with >250 employees globally were excluded. Within the four local authorities, individual outlets belonging to each identified large business (individual businesses could contribute to multiple outlets, e.g. chain restaurants) were identified using Ordnance Survey Points of Interest data from September 2020. Following this, we used stratified random sampling by business type and IMD quintile within each local authority to select outlets for inclusion. Business types were categorised by Ordnance Survey as follows: restaurants; pubs and bars; retail; hotels; cafes; fast food; attractions, and entertainment. A total of 330 outlets were sampled for data collection for pre and post time points and approximately 10 participants were recruited to take part in the study from each outlet at both pre and post data collection time points. This gave a total sample of 6578 customers from 330 outlets. This area sampling methods was utilize to recruit a varied sample of participants from different socioeconomic backgrounds and areas of England.<br><br>The mean age of participants was 41 years old (SD=18.7) pre implementation and 40.4 years old (SD=17.9) post implementation. Pre implementation 51% of the sample was male and at post implementation 47% of the sample was male. Pre implementation 64% of

people were from a low SEP background and post implementation 52% of people were from a low SEP. background The majority of the sample (84% pre implementation and 82% post implementation) were from a white ethic background.

Sampling strategy

The sampling procedure was opportunity sampling. The sample size required for customer exit surveys was based on results from a Cochrane review which included 28 studies examining the effect of nutritional labelling on purchasing and consumption30. The sample size was calculated to detect a 47kcal reduction from a baseline mean of 706kcal (SD 326) purchased per individual (7% reduction) as reported in the Cochrane review. Assuming a modest intra-class correlation of kcals purchased within outlets of 0.3935 and 10 participants per outlet, we estimated required sample sizes of N=3440 at pre and post from 344 outlets to detect a 7% reduction in energy purchased or consumed per participant with 80% power at α=0.05.

Data collection

Exit surveys with customers from sampled outlets were conducted to measure the number of kcals purchased and consumed, kcal knowledge of meal purchases, and self-reported noticing and use of kcal labelling. Researchers stood outside the selected food outlets during peak operating times (typically 12 pm – 9 pm, Wednesday to Sunday) and recruited customers as they entered or exited the outlet. Participants completed a short exit survey. Basic demographic information was collected (age, gender, ethnicity, and highest education level) with education level used to indicate participants' socioeconomic position (SEP) (lower SEP=school level qualifications or lower; and higher SEP= post-school level qualifications). Participants were asked to estimate the total number of kcals in their purchases. Following this, participants were asked about whether they noticed kcal labelling provided by the outlet (yes/no), whether they used this when making their purchases (yes/no) and if yes, why (to select lower kcal options, to select higher kcal options, other) and how (selected alternative meal option, selected a smaller or larger portion, made a meal substitution or customisation). Participants were then asked to report the food and drink items that they purchased from the outlet for their own consumption and to estimate any food that was shared or was not consumed. Self-reporting of shared items and leftovers was used to calculate consumption values for each participant. Whenever possible, customers were asked to provide a receipt to verify purchases, however, many outlets were not issuing receipts during data collection due to hygiene concerns and procedural changes related to the COVID-19 pandemic. Data was recorded using electronic tablets.
Exit surveys with customers from sampled outlets were conducted to measure the number of kcals purchased and consumed, kcal knowledge of meal purchases, and self-reported noticing and use of kcal labelling. Researchers stood outside the selected food outlets during peak operating times (typically 12 pm – 9 pm, Wednesday to Sunday) and recruited customers as they entered or exited the outlet. Participants completed a short exit survey. Basic demographic information was collected (age, gender, ethnicity, and highest education level) with education level used to indicate participants' socioeconomic position (SEP) (lower SEP=school level qualifications or lower; and higher SEP= post-school level qualifications). Participants were asked to estimate the total number of kcals in their purchases. Following this, participants were asked about whether they noticed kcal labelling provided by the outlet (yes/no), whether they used this when making their purchases (yes/no) and if yes, why (to select lower kcal options, to select higher kcal options, other) and how (selected alternative meal option, selected a smaller or larger portion, made a meal substitution or customisation). Participants were then asked to report the food and drink items that they purchased from the outlet for their own consumption and to estimate any food that was shared or was not consumed. Self-reporting of shared items and leftovers was used to calculate consumption values for each participant. Whenever possible, customers were asked to provide a receipt to verify purchases, however, many outlets were not issuing receipts during data collection due to hygiene concerns and procedural changes related to the COVID-19 pandemic. Data was recorded using electronic tablets.
Exit surveys with customers from sampled outlets were conducted to measure the number of kcals purchased and consumed, kcal knowledge of meal purchases, and self-reported noticing and use of kcal labelling. Researchers stood outside the selected food outlets during peak operating times (typically 12 pm – 9 pm, Wednesday to Sunday) and recruited customers as they entered or exited the outlet. Participants completed a short exit survey. Basic demographic information was collected (age, gender, ethnicity, and highest education level) with education level used to indicate participants' socioeconomic position (SEP) (lower SEP=school level qualifications or lower; and higher SEP= post-school level qualifications). Participants were asked to estimate the total number of kcals in their purchases. Following this, participants were asked about whether they noticed kcal labelling provided by the outlet (yes/no), whether they used this when making their purchases (yes/no) and if yes, why (to select lower kcal options, to select higher kcal options, other) and how (selected alternative meal option, selected a smaller or larger portion, made a meal substitution or customisation). Participants were then asked to report the food and drink items that they purchased from the outlet for their own consumption and to estimate any food that was shared or was not consumed. Self-reporting of shared items and leftovers was used to calculate consumption values for each participant. Whenever possible, customers were asked to provide a receipt to verify purchases, however, many outlets were not issuing receipts during data collection due to hygiene concerns and procedural changes related to the COVID-19 pandemic. Data was recorded using electronic tablets.

Timing

Data was collected between August-December 2021 for the pre policy data collection time point and August-November 2022 for the post-policy data collection time point, approximately 6 months after the policy was implemented.

Data exclusions

If the total number of kcals the participant purchased was unavailable or incomplete, they were excluded from the kcal purchased, kcal consumed, and kcal estimates primary analyses. However, these participants were retained for the analyses of noticing and use of kcal labelling. The number of exclusions is reported by reason for missing data in the supplementary materials.

Non-participation

Due to the sampling procedure used in this study we do not have data to report on how many participants declined participation however no participants dropped out of the study.

Randomization

This was a real-world observation study that did not included randomisation.

Reporting for specific materials, systems and methods

We require information from authors about some types of materials, experimental systems and methods used in many studies. Here, indicate whether each material, system or method listed is relevant to your study. If you are not sure if a list item applies to your research, read the appropriate section before selecting a response.

Materials & experimental systems	
n/a	Involved in the study
☒	☐ Antibodies
☒	☐ Eukaryotic cell lines
☒	☐ Palaeontology and archaeology
☒	☐ Animals and other organisms
☒	☐ Clinical data
☒	☐ Dual use research of concern
☒	☐ Plants

Methods	
n/a	Involved in the study
☒	☐ ChIP-seq
☒	☐ Flow cytometry
☒	☐ MRI-based neuroimaging

Plants

Seed stocks: N/A

Novel plant genotypes: N/A

Authentication: N/A
